# Supplementary material for: Recent Advances in Pyrimidine-Based Drugs
Source: Pharmaceuticals (Basel). 2024 Jan 11;17(1):104. doi: 10.3390/ph17010104 (PMC10820437; doi:10.3390/ph17010104)

Recent Advances in Pyrimidine-Based Drugs

Baskar Nammalwar [1] and Richard A. Bunce [2,*]

[1] Affiliation 1: nbaskarphd@gmail.com (Vividion Therapeutics, 5820 Nancy Ridge Drive, San Diego, CA 92121, USA)
[2] Affiliation 2: Department of Chemistry, Oklahoma State University, Stillwater, OK 74078-3071, USA
* Correspondence: rab@okstate.edu; Tel.: +1-405-744-5952

Comparison compounds mentioned in this article (in order of appearance).

Certinib - antitumor drug for anaplastic lymphoma kinase (ALK)

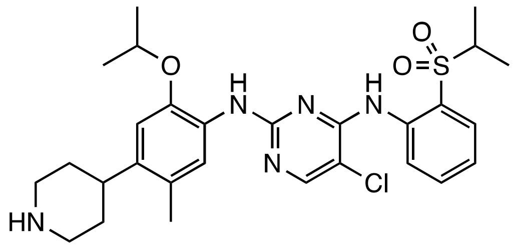

Isoniazid - antibiotic for treating tuberculosis

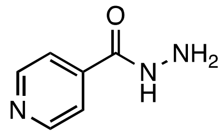

Rifampicin - antibiotic used to treat bacterial infections such as tuberculosis

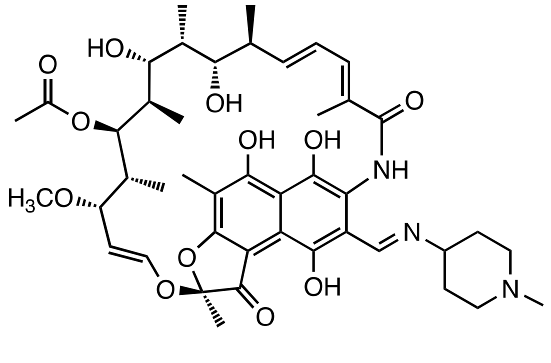

Linezolid - antibiotic used to treat Gram positive infections

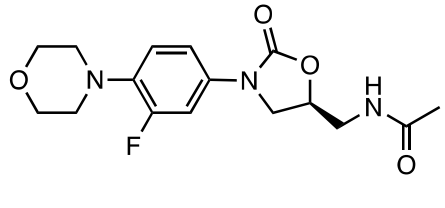

Methicillin - a penicillinase-resistant β-lactam antibiotic related to penicillin

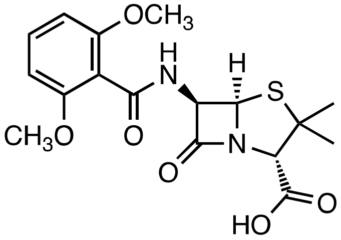

Amphotericin B - an antifungal agent used for fungal infections and leishmaniasis

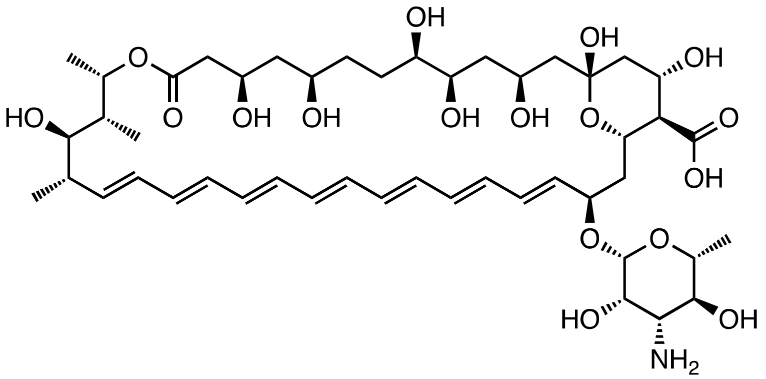

Fluconazole - an antifungal medication

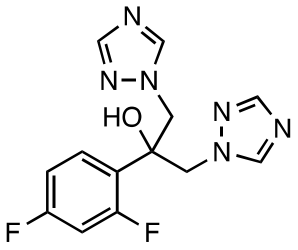

BH-11C - and NNRTI antiviral active against HIV-1

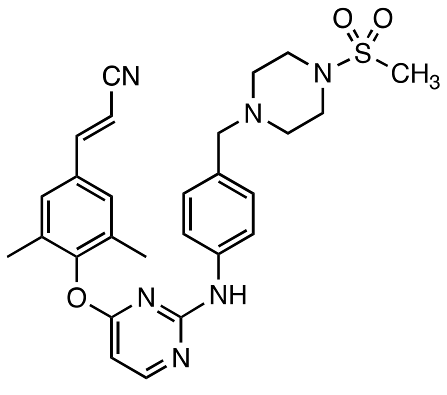

Oseltamivir (Tamiflu®) - an antiviral used to treat influenza A and B

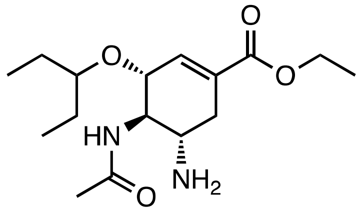

Baloxavir (Xofluza®) - an antiviral used to treat influenza A and B

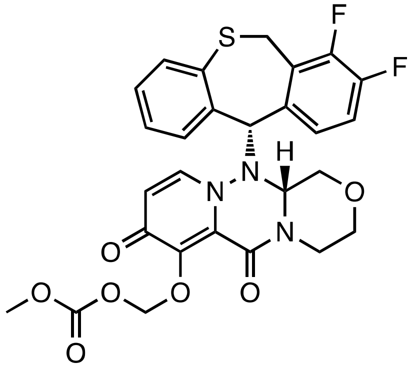

TAE226 - a dual inhibitor of focal adhesion kinase and insulin-like growth factor-I receptor

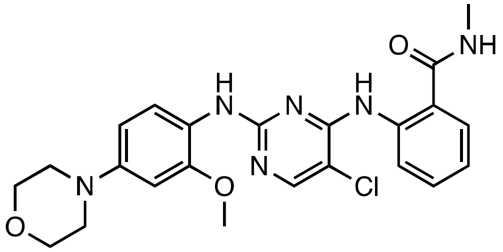

5-Fluorouracil - a cytotoxic chemotherapy medication used against cancer

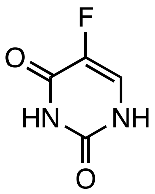

Staurosporine - an alkaloid antibiotic from *Streptomyces staurosporeus*; an ATP protein kinase inhibitor

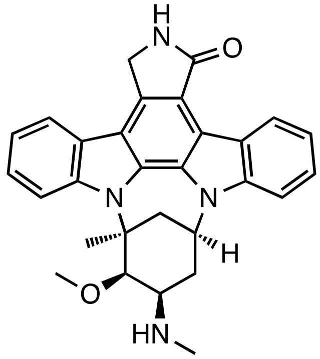

Cetuxiab (Erbitux®) - a recombinant monoclonal antibody; an epidermal growth factor inhibitor medication used to treat metastatic cancers

Pantitumumab (Vectibix®) - a human monoclonal antibody specific to the EGFR, Erb-1, HER1 receptor in humans

Necitumumab - a recombinant human IgG1 monclonal antibody used in the treatment of tumors and malignant cells

Neratinib (Nerlynx®) - a tyrosine kinase inhibitor used in the treatment of breast cancer

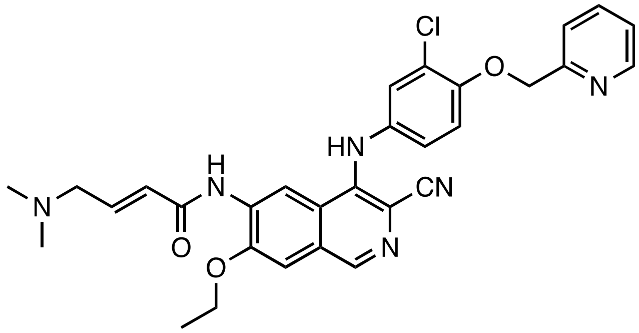

Gefitinib (Iressa®) - therapeutic agent used to treat various breast, lung and other cancers

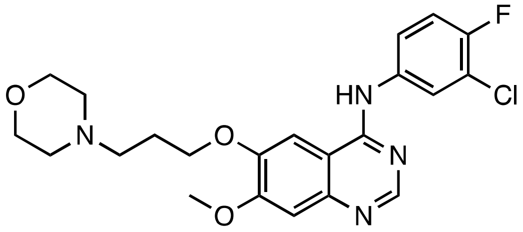

Erlotinib (Tarceva®) - agent used to treat non-small cell lung cancer

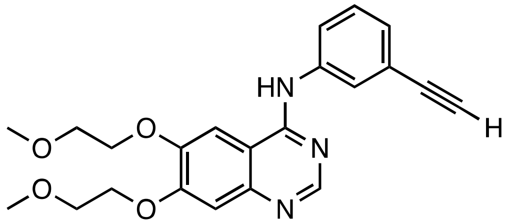

Lapatinib (Tykerb®) - a dual tyrosine kinase inhibitor which interrupts the EGFR and HER2 pathways used for treating breast cancer

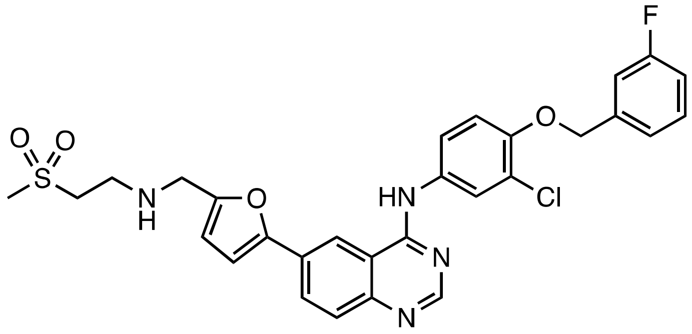

Afatinib (Gilotrif®) - a tyrosine kinase inhibitor medication used to treat non-small cell lung cancer

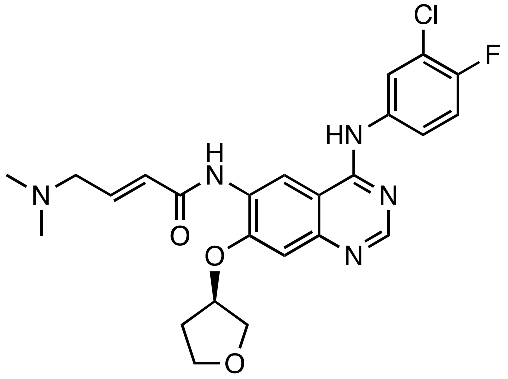

Vandetinib (Caprelsa®) - a medication used to treat thyroid cancer

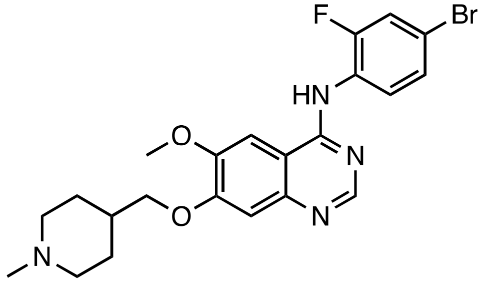

Brigatinib (Alunbrig®) - an anaplastic lymphoma kinase (ALK) and epidermal growth factor receptor (EGFR) inhibitor used to treat cancer

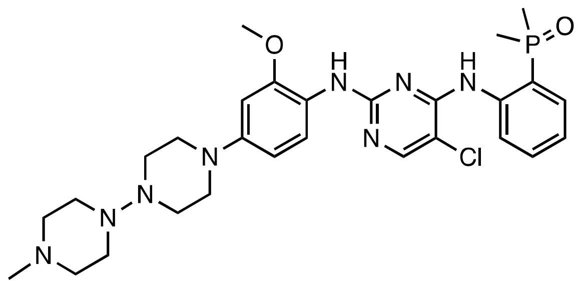

Abemaciclib - a CDK inhibitor medication for treatment of advanced metastatic beast cancers

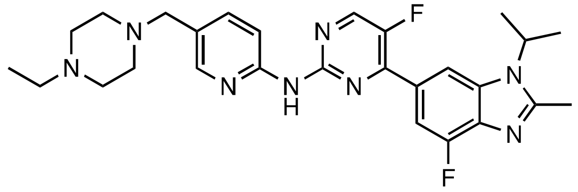

Colchicine - a medication used to treat gout and Behçet's disease (an inflammatory disorder of the eye)

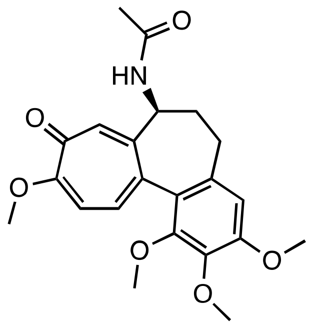

Paclitaxel (Taxol®) - used to treat ovarian, esophageal, breast, lung cervical and pancreatic cancers

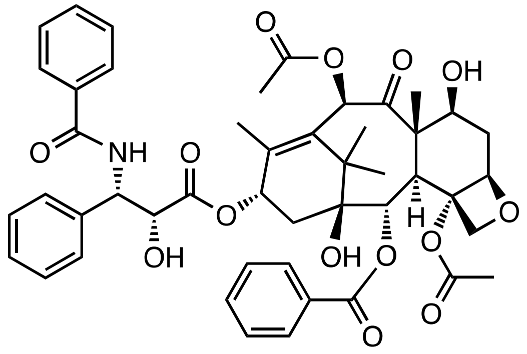

GSK-J1 - an H3K27 histone demethylase inhibitor anticancer agent

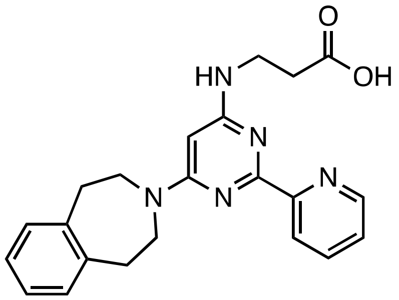

Cytarabine - medication to treat acute myeloid leukemia, chronic myelogenous leukemia and acute lymphoblastic leukemia

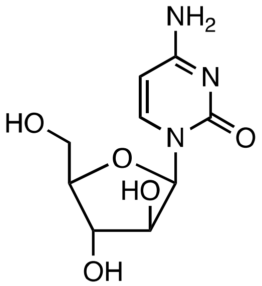

Daunorubicine (aka Daunomycin) - medication to treat acute myeloid leukemia, chronic myelogenous leukemia and acute lymphoblastic leukemia

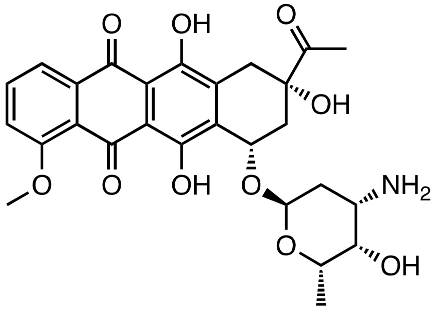

Sorafenib (Nexavar®) - a kinase inhibitor used to treat kidney and liver cancer as well as FMS-like tyrosine kinase 3- internal tandem duplications (FLT3-ITD) positive AML

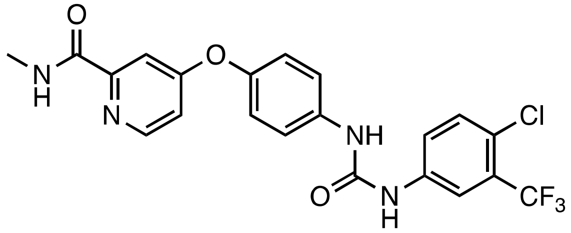

Enzalutamide (Xtandi®) - a non-steroidal antiandrogen used to treat prostate cancer

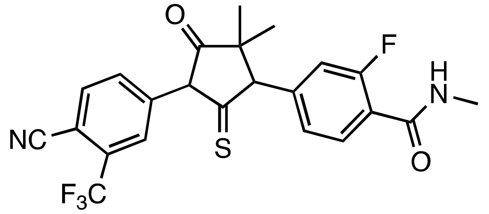

HG-9-91-01 - a pan-SIK inhibitor of necroptosis

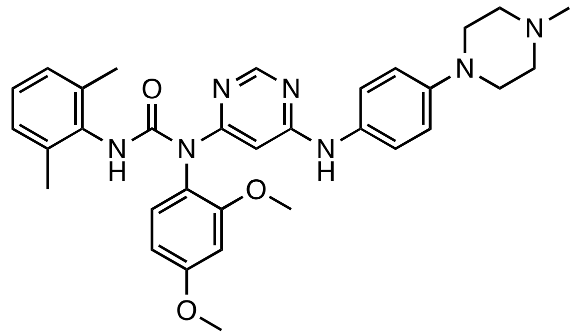

Dasatinib (Sprycel®) - medication to treat chronic myelogenous leukemia and acute lymphoblastic leukemia

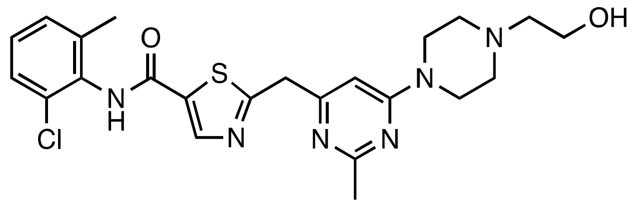

Fedratinib (Inrebic®) - an anticancer medication to treat myeloproliferative disease

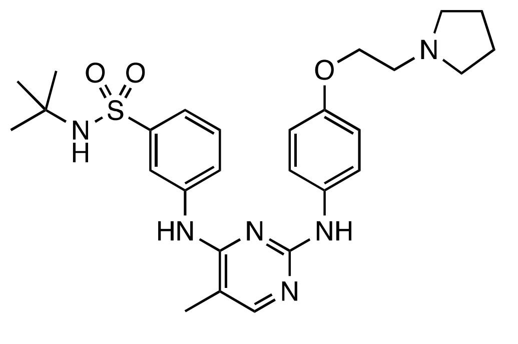

Dexamethasone - a corticosteroid that prevents the release of substances in the body that cause inflammation

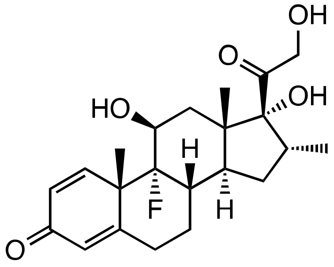

Pioglitazone (Actose®) - an anti-diabetic medication used to treat type 2 diabetes

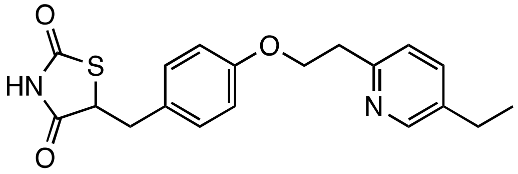

Supplement: Supplementary file 1 [file pharmaceuticals-17-00104-s001.zip › pharmaceuticals-2767510-supplementary.pdf]
